# Assessment of Metalloproteinase Matrix 9 (*MMP9*) Gene Polymorphisms Risk Factors for Pelvic Organ Prolapse in the Brazilian Population

**DOI:** 10.1055/s-0039-1681112

**Published:** 2019-04-02

**Authors:** Frederico Rezende Ghersel, Ricardo Peres Souto, Ester Wilma Pacheco Gonzales, Denise Souza Paulo, César Eduardo Fernandes, Emerson Oliveira

**Affiliations:** 1 Department of Urogynecology and Vaginal Surgery, Discipline of Gynecology, Faculdade de Medicina do ABC, Santo André, SP, Brazil

**Keywords:** polymorphism, matrix metalloproteinase, pelvic organ prolapse, polimorfismo, matriz de metaloproteinase, prolapso de órgão pélvico

## Abstract

**Objective** To evaluate the *C-1562T* matrix metalloproteinase 9 (*MMP9*) gene polymorphisms as risk factors related to the occurrence of pelvic organ prolapse (POP) and to identify the clinical variables associated with the occurrence of the disease. Epidemiological studies of risk factors for POP do not explain why nulliparous women with no known risk factors also develop POP. Therefore, genetic factors may be involved.

**Methods** Cohort study with 86 women with symptomatic POP (cases), and 158 women without a prior or current diagnosis of this disorder (controls). The groups were analyzed for the presence of *MMP9* gene polymorphisms. Genotyping was performed using polymerase chain reaction (PCR) with DNA obtained from a peripheral venous puncture of both groups.

**Results** There were no differences between the cases and controls even when we grouped the mutant homozygous and heterozygous genotypes. The analysis of patients with a complete absence of POP versus patients with total POP also showed no statistically significant differences. Age and home birth were found to be independent risk factors for POP.

**Conclusions** There were no statistically significant differences in the *C-1562T* MMP9 polymorphisms between the cases and controls in Brazilian women.

## Abstract (pt)

**Resumo:**

**Objetivo** Avaliar polimorfismos do gene *C-1562T* do gene matriz de metaloproteinase 9 (*MMP9*) como fatores de risco relacionados à ocorrência de prolapso de órgão pélvico (POP) e identificar variáveis clínicas associadas à ocorrência da doença. Estudos epidemiológicos de fatores de risco para POP não explicam por que mulheres nulíparas sem fatores de risco conhecidos também desenvolvem POP. Portanto, fatores genéticos podem estar envolvidos.

**Métodos** Estudo de coorte com 86 mulheres com POP sintomático (casos) e 158 mulheres sem diagnóstico prévio ou atual deste transtorno (controles). Os grupos foram analisados quanto à presença de polimorfismo do gene *MMP9*. A genotipagem foi realizada por reação em cadeia da polimerase (RCP) com DNA obtido por punção venosa periférica dos indivíduos em ambos os grupos.

**Resultados** Não houve diferenças entre os casos e controles, mesmo quando agrupamos os genótipos mutantes homozigotos e heterozigotos. A análise de pacientes com ausência completa de POP *versus* pacientes com POP total também não mostrou diferenças estatisticamente significativas. Idade e parto domiciliar foram considerados fatores de risco independentes para o POP.

**Conclusão** Não houve diferenças estatisticamente significativas no polimorfismo *C-1562T* do gene *MMP9* entre os casos e controles em mulheres brasileiras.

## Introduction

In its latest publication, the International Urogynecological Association (IUGA)/International Continence Society (ICS) considered pelvic organ prolapse (POP), primarily as an anatomical change of pelvic organs, such as the uterus and/or the different vaginal compartments and their neighboring organs, such as the bladder, the rectum, or the bowel.^1^ Pelvic organ prolapse is a complex entity with a multifactorial etiology and various predisposing factors that include pregnancy, vaginal births, advanced age, skeletal structure variation, neuromuscular involvement, congenital factors, genetic factors, ethnic factors, body mass index (BMI), and connective tissue diseases.^2^ The estimated prevalence of POP is of 21.7% in women aged between 18 and 83 years old, and reaches 30% in patients aged between 50 and 89 years old.^3^ Estimates indicate that Brazil will have ∼ 9 million women > 80 years old in 2050. Given the aging of the population, the high morbidity caused by genital prolapse, and the high cost of treatment, the country will face a major public health problem.^4^

Pelvic organ prolapse is negatively related to quality of life (QoL) because it affects daily activities, self-image, and sexual relations.^5^ Additionally, symptomatic prolapses result in a decreased QoL due to discomfort and urinary and/or intestinal dysfunctions.^1^ ^5^ The risk factors for POP are traditionally considered to be obstetrical factors, aging, obesity, diabetes, and postmenopausal estrogen deficiency.^6^ However, nulliparous women without any risk factors can also develop POP. Therefore, genetic factors may play a role in the genesis of this disease. When a mother has POP, the relative risk for the daughter to develop the disease is 3.2-fold. Additionally, the risk of a woman developing POP when she has a sister with the disease is 2.4-fold.^7^ The future of genital prolapse treatment will be influenced by genetics, biological pelvic changes, changes in tissue homeostasis, and topical hormones, rather than general pelvic anatomical surgical correction.^8^ Epidemiological evidence suggests that some women have a genetic predisposition for the development of urogenital prolapse and stress urinary incontinence (SUI). The abnormal expression of multiple structural proteins is thought to be the molecular genetic mechanism underlying the development of these conditions. For instance, a group of families with an autosomal dominant pattern of POP transmission with high penetrance was identified.^9^ The changes in the pelvic floor due to all of the etiological factors vary individually and seem to determine the occurrence of POP only in women with a predisposition for its development.^10^ Biochemical or structural defects of the components supporting the pelvic floor could contribute to the development of the disease.^11^

Metalloproteinases are capable of degrading collagen and other extracellular matrix components. Matrix metalloproteinases (MMPs) are a family of extracellular endopeptidases that are defined based on the presence of conserved prodomains and catalytic domains.^12^ Matrix metalloproteinase 1(MMP1) plays an important role in the degradation of collagen type I, whereas MMP3 is able to activate other MMPs, including MMP1.^13^ Vulic et al^14^ measured the expression of collagen type I and MMP1 in uterosacral ligaments of postmenopausal women, comparing women with POP with control subjects. The women with POP revealed a significantly lower expression of collagen I and a significantly higher expression of MMP1 compared with the control subjects. The molecular mechanism involved in the aforementioned findings remains unknown, but may involve single nucleotide polymorphisms in the regulatory areas of genes that encode proteins relevant to connective tissue functions. These polymorphisms change collagen patterns and *MMP* gene expression.^13^

Matrix metalloproteinase 9 is a protease that has been associated with the degradation of both collagen and elastin in the extracellular matrix.^15^ In humans, several studies have shown that the activity of MMP9 is increased in the vaginal tissue in women with POP when compared with women without POP.^16^

Changes in the genes that encode MMP9 could interfere with the genesis of POP; thus, the objective of the present study was to evaluate polymorphisms of the *C-1562T MMP9* gene as a risk factor related to the occurrence of female genital prolapse. The accurate identification of patients with an increased risk for the development of POP would be revolutionary for the prevention of this condition. This advance could influence the choice of the best delivery route. For instance, there is a great debate among researchers regarding the risks and benefits of elective cesarean section and normal delivery in terms of the damage caused to the pelvic and perineal structures.^17^ Furthermore, accurate identification would allow a better assessment of candidate patients for vaginal corrective surgery of genital prolapses by identifying those with a greater risk of recurrence.^18^

The aim of the present study was to evaluate the functional *C-1562T MMP9* gene polymorphism as a risk factor related to the occurrence of POP, a single nucleotide polymorphism located in the promoter region of the *MMP9* gene.

## Methods

The present study was conducted in the Urogynecology and Vaginal Surgery Sector of the Department of Gynecology and Obstetrics and in the laboratory of the Biochemistry Program of the Faculdade de Medicina do ABC (FMABC, in the Portuguese acronym), Santo André, state of São Paulo, Brazil. To ensure the rights and duties related to the scientific community, to the study subjects, and to the State, the present study complied with the guidelines of the Resolution 196/96 of the Brazilian National Health Council (CNS, in the Portuguese acronym), and was previously submitted for evaluation and approved by the Research Ethics Committee of the FMABC under the number 554.670/2014. All of the patients were informed about the study and signed a consent form for participation in the study. This is a cohort study that was part of a regular project and received financial support from the Fundação de Amparo à Pesquisa do Estado de São Paulo (FAPESP, in the Portuguese acronym) under the number 2014/01107–6. We have conducted a physical examination, supplementary routine tests, and blood sample collection for the analysis of the proposed genetic polymorphisms.

A total of 86 women with POP and 158 women without POP were recruited. The inclusion criteria were postmenopausal patients with genital dystopia stages III and IV (according to the POP quantification system [POP-Q] of the ICS) and the absence of associated neurological diseases. The exclusion criteria were patients with sequelae from high exposure to ionizing radiation, pregnant women, malignant gynecological diseases, connective tissue diseases (rheumatoid arthritis, lupus, scleroderma, polymyositis, dermatomyositis, Marfan syndrome, or Ehler-Danlos syndrome), patients who declined to participate in the study, and losses to follow-up.

The control group was composed of postmenopausal patients with a gynecological examination of the external genitals that showed at most genital prolapse stage I. The groups were analyzed for the presence of polymorphism of the *C-1562T MMP9* gene. The genotyping was conducted via polymerase chain reaction (PCR) with DNA collected from a peripheral venous puncture of both the control and case groups.

**Fig. 1:**
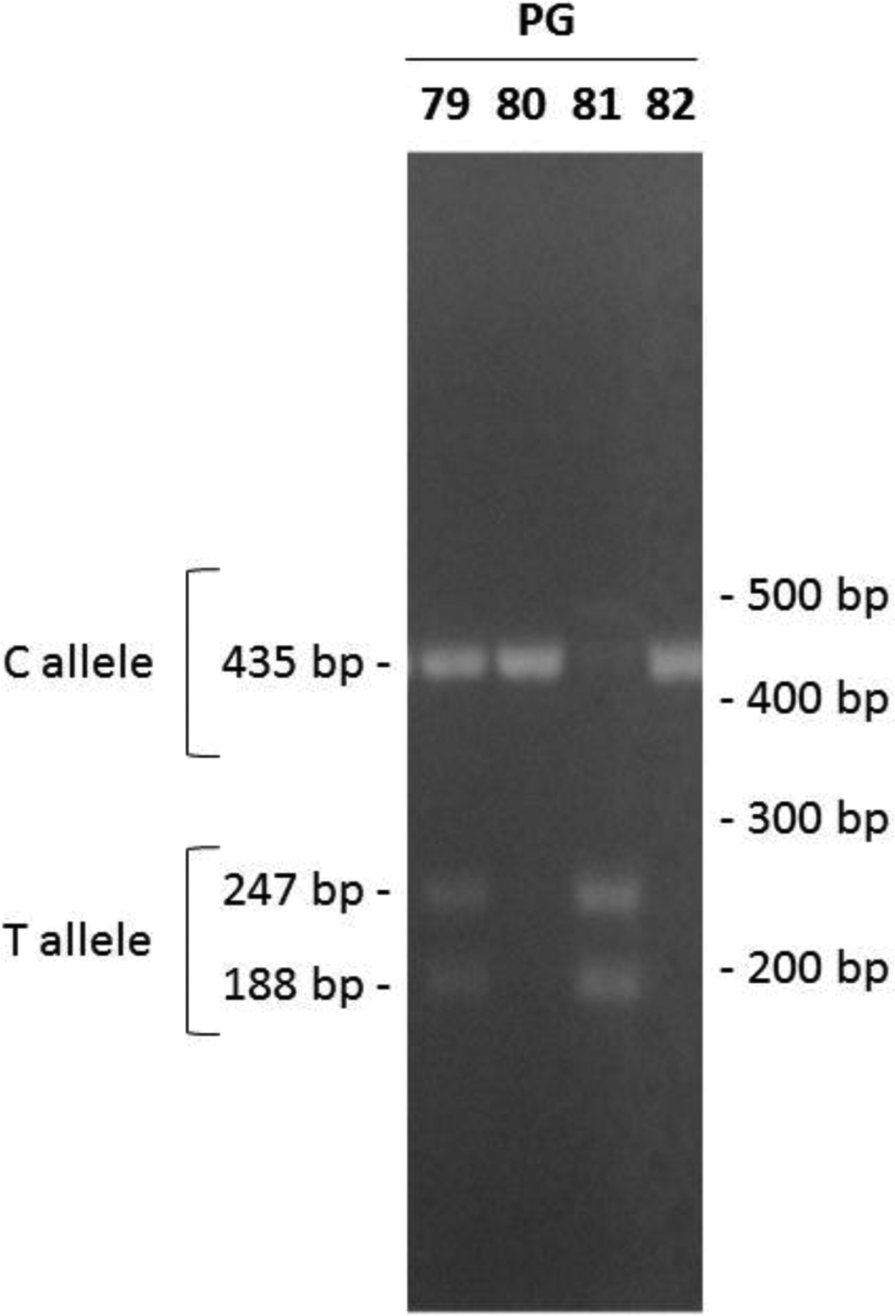
Agarose gel electrophoresis.

The following equipment was used for the genomic DNA extraction, PCR amplification and polymorphism analysis by restriction fragment length polymorphism (RFLP): a tube shaker Vortex Genie 2 (Fisher Scientific, Bohemia, NY, USA), a microtube centrifuge 5415C (Eppendorf), a clinical centrifuge Excelsa 2206 (Fanem, Rio de Janeiro, RJ, Brazil), a deionizer Elix 100 (Millipore, Molsheim, France), a UV spectrophotometer Nanoview Plus (GE Healthcare, Chicago, IL, USA), micropipettes Pipetman (Gilson, Villiers-le-Bel, France), a thermocycler Mastercycler Personal (Eppendorf, Hamburg, Germany), a UV transilluminator ZT-21 (LGC Biotecnologia, Cotia, SP, Brazil) and an electrophoresis power supply EPS301 (Amersham Biosciences, Little Chalfont, UK). The reagents used were included in the Illustra-Blood genomicPrep Mini Spin DNA extraction kit (GE Healthcare, Chicago, IL, USA). The PCR technique used nuclease-free water, the Master Mix 2x concentrate (Promega, Madison, WI, USA) and the primers proposed by Zhang et al,19 which were synthesized by DNA Express Biotechnology (Guarulhos, SP, Brazil) (forward: 5-GCCTGGCACATAGTAGGCCC-3′ and reverse: 5′-CTTCCTAGCCAGCCGGCATC-3′). Agarose gel electrophoresis was used to verify the presence of genomic DNA in the extractions. In the PCR amplification, a pair of primers was used to amplify the 435-bp sequence found in the promoter region (-1809 to - 1374) of the *MMP9* gene.^20^ After the PCR amplification was finished, agarose gel electrophoresis was used to verify that the amplification was successful. The samples amplified by PCR were digested with the SphI restriction enzyme. This enzyme cleaves the T allele at the polymorphic position-1562 of the *MMP9* promoter to generate 2 fragments with 247 bp and 188 bp. The DNA fragments were separated by 100 V electrophoresis until the fractions reached the gel border. The DNA bands were viewed by fluorescence in a UV transilluminator (Amersham Biosciences, Little Chalfont, UK) (Fig. 1).

The normality of the quantitative data was verified using the Shapiro-Wilk test. We have compared the qualitative variables using the Chi-squared and the Fisher exact tests. An unpaired *t*-test was used for the quantitative variables. The data were analyzed using GraphPad Prism 6 (GraphPad Software, San Diego, CA, USA) and IBM SPSS Statistics for Windows, Version 23.0 (IBM Corp., Armonk, NY, USA). After the stratification of the groups, the influence of some clinical characteristics on the POP risk was estimated using the odds ratios (ORs) obtained from the binary logistic regression model. The adopted significance level was 5% (*p* < 0.05), and the adopted confidence interval (CI) was 95%.

## Results

The genic balance analysis conducted using the Chi-squared test revealed that the Hardy-Weinberg principle was met (*p* = 0.275, in which *p* < 0.05 is not consistent with the Hardy-Weinberg equilibrium and there is no accuracy if < 5 individuals are included). As shown in Table 1, we have observed no differences between the cases and controls when the groups were compared, even when we grouped the mutant homozygous (TT) and heterozygous genotypes (CT). To verify whether *C-1562T MMP9* polymorphism could be responsible for more severe genital prolapse cases, we have stratified the assessment and analyzed patients with a complete absence of prolapse (stage 0) versus patients with total prolapse (stage IV). As shown in Table 2, we did not observe significant differences. Finally, the study design included the evaluation of clinical characteristics as possible risk factors for the occurrence of POP (Table 1). Table 3 presents the crude and adjusted ORs of these characteristics. By multivariable logistic regression analyses, we have found that age (OR = 11.89; CI = 3.53–40; *p* < 0.0001), and home birth (OR = 9.645; CI = 3.35–27.7; *p* < 0.0001) were significantly associated with POP.

**Table 1:** Analysis of the clinical features and matrix metalloproteinase-9 gene polymorphisms of cases and controls

Variables	Cases (Mean, median, or %)	Controls (Mean, median, or %)	*p*-value
Age (years)	68.4	57.8	< 0.0001^a^
Ethnicity			0.422^b^
White	69.9%	64.8%	
Not White	30.1%	35.2%	
Body mass index (kg/m^2^)	28.8	28.9	0.874^a^
Age of menopause	48.8	46.6	0.07^a^
Hormonal therapy	10.7%	18.1%	0.09^b^
Smoking	13.1%	20.1%	0.15^b^
Arterial hypertension	57.8%	49.4%	0.186^b^
Diabetes mellitus	24.5%	23.7%	0.888^b^
Dyslipidemia	25.4%	24.7%	0.889^b^
Chronic cough	1.8%	6.8%	0.08^b^
Intestinal constipation	14.3%	10.4%	0.35^b^
Pregnancy	4 (1–24)	3 (0–16)	< 0.0001^a^
Parity	4 (1–16)	3 (0–15)	< 0.0001^a^
Vaginal delivery	3 (0–16)	2 (0–15)	< 0.0001^a^
Cesarean section	0 (0–1)	0 (0–3)	0.377^a^
Heaviest birthweight (g)	3,516	3,059	0.147^a^
Episiotomy	8.3%	9.2%	> 0.999^b^
Labor analgesia	3.7%	4.8%	0.768^b^
Home birth	25.9%	3.05%	< 0.0001^b^
Hysterectomy	15.2%	15.6%	> 0.999^b^
Exaggerated physical exercise	22.5%	14.1%	0.077^b^
***MMP9*** **genotypes**			0.2749^c^
GG	73 (84.8%)	126 (79.7%)	
TG	13 (15.2%)	28 (17.7%)	
TT	0%	4 (2.6%)	
**Grouped ** ***MMP9*** **genotypes**			0.3890^b^
GG	73 (84.8%)	126 (79.7%)	
TG + TT	13 (15.2%)	32 (20.3%)	

Abbreviations: GG, wild homozygous; *MMP9*, matrix metalloproteinase-9; TG, heterozygous; TT, polymorphic homozygous.

a Unpaired *t*-test.

b Fisher exact test.

c Chi-squared test.

**Table 2:** Genotypic distribution between patients with a complete absence of prolapse versus patients with prolapse stage IV

Genotypes	POP stage IV (*n* = 33)	POP stage 0 (*n* = 105)	*p*-value
GG	27 (81.8%)	82 (78.10%)	0.807^a^
TT + CT	6 (18.2%)	23 (21.90%)	

Abbreviations: GG, wild homozygous; POP, pelvic organ prolapse; TT + CT, mutant homozygous and heterozygous genotypes.

a Fisher exact test.

**Table 3:** Odds ratios (crude and adjusted) for the occurrence of pelvic organ prolapse

Covariate	Crude OR (CI)	*p-value*	Adjusted OR (CI)	*p*-value
Age ≥ 51	15.57 (4.73–51.2)	< 0.0001	11.89 (3.53–40)	< 0.0001
Pregnancy ≥ 3	2.02 (1.24–3.28)	0.004	0.656 (0.283–1.51)	0.325
Vaginal delivery ≥ 3	3.12 (1.86–5.23)	< 0.0001	1.91 (0.7–5.22)	0.202
Parity ≥ 3	2.64 (1.62–4.31)	< 0.0001	2.01 (0.665–6.1)	0.216
Home birth	11.1 (4.14–29.7)	< 0.0001	9.645 (3.35–27.7)	< 0.0001

Abbreviation: CI, confidence interval.

## Discussion

Although current knowledge on the genetic susceptibility to POP is limited, research in this field is increasing. Pelvic organ prolapse may be considered a common and complex disease for which there are multiple potential susceptibility markers.^5^

In agreement with the premise that there is a genetic predisposition for the occurrence of POP, Lammers^21^ showed that women with POP were more likely to have first- and second-degree relatives with this condition.

Genetic epidemiological data concerning the *MMP9* gene and the occurrence of advanced POP are limited, especially in the non-Hispanic population.^15^ Nevertheless, some studies compared the *MMP9* activity in the vaginal tissues of women with and without a genital prolapse and found an increase in *MMP9* activity in association with this disease.^20^ ^22^

In our review, 1 study that aimed to establish a relationship between the *rs17576* and *rs3918242* MMP9 polymorphisms and the risk of POP was performed by Chen et al,^23^ which included 92 Chinese women with POP and 152 women without the disease. The authors observed, after a binary logistic regression, that the *MMP9 rs17576* polymorphism seemed to be associated with POP, but did not find any association between the *MMP9 rs3918242* polymorphism and the risk of POP, which is in line with the present study.

It should be emphasized that our results may not be extrapolated to other ethnic groups. We must consider the miscegenation of the Brazilian population and, therefore, our results may be different from the group of the Chinese authors aforementioned.

Although the case and control groups were not completely homogeneous, a logistic regression model was applied to eliminate confounding factors and to identify independent risk factors. Our study showed that age, parity, vaginal delivery, home delivery, and number of pregnancies were significantly different between the groups. However, the multivariate logistic regression analysis identified only age and home birth as independent risk factors for the occurrence of POP, which are already well-known as risk factors in the literature.^24^ ^25^ ^26^ Other known risk factors, such as parity, vaginal delivery, and number of pregnancies, were not confirmed, probably due to the small sample size.

The design of the present study has limitations, since the case and control groups differ significantly in age and home birth – both risk factors of POP. Besides, the sample size was relatively small, and our negative results may represent false-negative findings. Thus, a larger well-designed study is needed to confirm a possible association.

It is important to consider the peculiar ethnic differences in the Brazilian population, and to understand that a sum of genetic changes is most likely necessary for the development of the disease. It is also important to consider that this is the first study to evaluate this polymorphism in the Brazilian population in association with the risk of POP. However, we believe that studies seeking to establish genetic markers for POP need to advance. These studies are especially important because the ability to identify individuals with an increased risk of developing POP via genetic screening can be useful in cases, such as in the decision for the most appropriate delivery route for large fetuses.

There is hope that focusing on the genetic susceptibility to POP will enable the stratification of the risk for women of developing the disease and thus establish prevention and lifestyle change strategies. These interventions could reduce the need for corrective surgery and improve the QoL of women who present the most serious stages of genital prolapse.^15^

## Learning Points

- Pelvic organ prolapse is a complex entity with a multifactorial etiology.
- Nulliparous women without any risk factors can also develop POP. Therefore, genetic factors may play a role in the genesis of the disease.
- Studies seeking to establish genetic markers for POP need to advance.
- Studies have shown that *MMP9* activity is increased in the vaginal tissue in women with POP compared with women without POP.
- *MMP9* polymorphisms seemed to be associated with POP in some populations

## Conclusion

Our results allow us to conclude that there are no differences in *C-1562T MMP9* polymorphisms between the cases and controls.
